# Upconversion Nanomaterials: Synthesis, Mechanism, and Applications in Sensing

**DOI:** 10.3390/s120302414

**Published:** 2012-02-23

**Authors:** Jiao Chen, Julia Xiaojun Zhao

**Affiliations:** Department of Chemistry, University of North Dakota, Grand Forks, ND 58202, USA; E-Mail: jiao.chen@my.und.edu

**Keywords:** upconversion nanoparticles, biosensing, NIR-radiation

## Abstract

Upconversion is an optical process that involves the conversion of lower-energy photons into higher-energy photons. It has been extensively studied since mid-1960s and widely applied in optical devices. Over the past decade, high-quality rare earth-doped upconversion nanoparticles have been successfully synthesized with the rapid development of nanotechnology and are becoming more prominent in biological sciences. The synthesis methods are usually phase-based processes, such as thermal decomposition, hydrothermal reaction, and ionic liquids-based synthesis. The main difference between upconversion nanoparticles and other nanomaterials is that they can emit visible light under near infrared irradiation. The near infrared irradiation leads to low autofluorescence, less scattering and absorption, and deep penetration in biological samples. In this review, the synthesis of upconversion nanoparticles and the mechanisms of upconversion process will be discussed, followed by their applications in different areas, especially in the biological field for biosensing.

## 1. Introduction

The upconversion phenomenon has been observed in transition metals, actinides, but mainly in the rare earth (RE) elements, which contain the lanthanide (Ln) series, yttrium, and scandium [1]. Ln^3+^ ions have special 4f^n^ 5d^0–1^ inner shell configurations that are well-shielded by outer shells and have abundant and unique energy level structures. These Ln^3+^ ions can exhibit sharp luminescence emissions via intra-4f or 4f-5d transitions. Their remarkable luminescence properties, such as narrow bandwidth, long-time emission, and anti-Stokes emission, have been widely applied in lasers, solar cells, analytical sensors, optical imaging, photodynamic therapy, and so on. At present, the low luminescence efficiency is one of the main limiting factors. Therefore, to obtain the highest upconversion luminescence efficiency, it is critical to choose an appropriate host material with lower phonon energy (high phonon frequencies of the host lattice lead to nonradiative relaxation). To date, host materials, including fluoride, chloride and bromide, have been shown to enhance upconversion luminescence intensity. Most of chlorides and bromides are sensitive to moisture, and thus are not suitable for labeling biomolecules (used mostly in aqueous solutions) [2]. RE fluorides, mainly REF_3_ and AREF_4_ (A = alkali), have been considered as an excellent host material due to their high refractive index and high transparency arising from low-energy phonons. These two advantages further lead to low probability of nonradiative decay and increased luminescence quantum yield.

Most fluorescent materials, including dye molecules, quantum dots, and dye-doped silica/gold nanomaterials, emit light by the downconversion process (emitting lower-energy photons under higher-energy irradiation). Although the uses of a conventional organic dye molecule or quantum dot (QD) based biomarker have achieved significant progress in real-time detection and bioimaging, they still have drawbacks. These fluorescent materials are generally excited by ultraviolet (UV) or visible light, which may induce autofluorescence and photodamage to biological samples, resulting in low signal-to-noise ratio and limited sensitivity. These limitations prompted the development of a new type of high-quality and well-shaped nanomaterials known as upconversion nanomaterials (UCNs). These UCNs usually consist of an inorganic host that is doped with Ln^3+^ ions. They show good biocompatibility and generally low cytotoxicity, and are in fact non-cytotoxic to a broad range of cell lines [3,4]. Furthermore, surface modification by ligand engineering [5–7], ligand attraction [8], surface polymerization [3,9–13], self-assembly [14–16] or layer-by-layer assembly technology [17], broadens their application fields. Surface modification by a silica shell is by far the most popular, common, and practical approach [3,11,18–20]. Proteins, DNA, biological macromolecules or other desirable targets can be easily linked to UCNs. In particular, the UCNs’ unique property of emitting visible light under NIR irradiation makes them a suitable candidate both for *in vivo* and *in vitro* bioimaging [21–23].

Theoretically, most lanthanide ions can undergo the NIR-to-visible upconversion process; however, relatively efficient upconversion is only possible with a few trivalent lanthanide ions (e.g., Er^3+^ and Tm^3+^) under low pump densities (980 nm excitation). Up to now, the most often-used upconversion nanoparticles are Yb^3+^-Er^3+^ or Yb^3+^-Tm^3+^ co-doped NaYF_4_ nanomaterials (NaYF_4_:Yb^3+^, Er^3+^ or NaYF_4_: Yb^3+^, Tm^3+^ UCNs). In this review, we will briefly discuss the mechanisms of upconversion phenomenon, but mainly focus on the recent progress in UCNs’ chemical syntheses and their applications in different areas, especially in the biological field.

## 2. Mechanism

Different upconversion luminescence mechanisms have been recognized either alone or in combination. Three basic mechanisms—excited state absorption (ESA), photon avalanche (PA), and energy transfer upconverion (ETU) (also known as *addition de photon par transferts d'energies*, APTE effect) will be discussed here.

### 2.1. Excited State Absorption (ESA)

**Figure 1.**
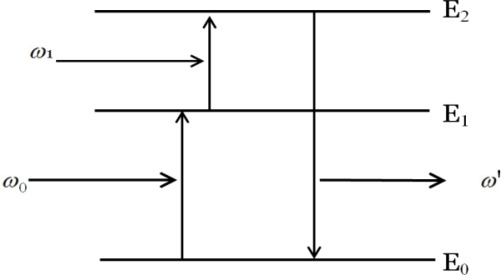
Schematic diagram of excited-state absorption (w’ > w_1_, w_0_). E_0_, E_1_, and E_2_ represent ground state, intermediate, and excited state, respectively. When one ion or electron in the E_0_ state absorbs one photon, it is first excited to the E_1_ state; after sequentially absorbing the second photon, it can jump to the excited state E_2_ and emit higher energy photons when coming back to the ground state.

ESA involves multistep excitation by sequentially absorbing one or more photons from the ground state to intermediate reservoir stage, and finally populates at excited state, from which upconverison luminescence occurs (Figure 1).

### 2.2. Photon Avalanche (PA)

**Figure 2.**
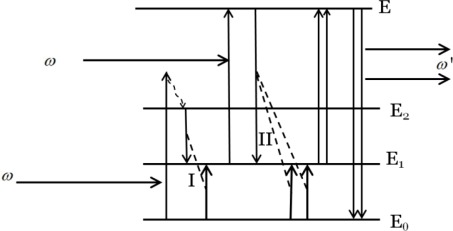
Schematic diagram of photon avalanche.

PA process is a more complex process, which can be characterized by three distinct nonlinear behaviors: transmission, emission, and rise time on the pump power intensity with generally the existence of a critical pump threshold. Take a four-energy system as an example (Figure 2), in which E_0_, E_1_ and E_2_, and E represents ground state, intermediate states, and upper excited states, respectively.

An electron or ion is excited when it absorbs the excitation radiation. The excitation radiation is usually not resonant with the absorption transition from the ground state to the intermediate states, but a little higher than E_2_. Through the cross relaxation, it goes down to E_2_ state. Energy transfer occurs between the E_2_ state electron and the E_0_ state electron, resulting in the formation of two electrons in the E_1_ state. One of them absorbs the excitation radiation and is excited to the E state, in which it interacts with E_0_ state electrons and energy transfer II occurs to form three E_1_ electrons. Here, the excitation radiation is resonant with the absorption transition from E_1_ to E. By repeating the whole steps again and again, the number of electrons in the E state increases dramatically. When the electrons go back to the E_0_ state, high-energy photons are emitted. In summary, the PA process basically involves resonant excited-state absorption, efficient cross relaxation and substantial population of the reservoir level, which leads to strong upconversion emission. Although PA is efficient, it still suffers from some disadvantages, such as limited maximum-output due to the weak ground–state absorption, and high pump powers needed to reach the threshold condition.

### 2.3. Energy Transfer Upconversion (ETU)

**Figure 3.**
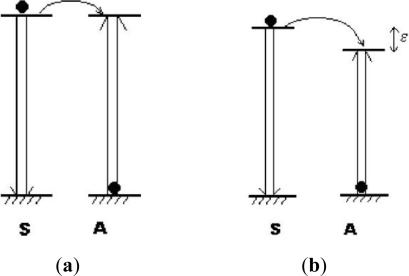
Energy transfer processes between two ions: (**a**) resonant non-radiative transfer; (**b**) phonon-assisted non-radiative transfer. (S: sensitizer ions, A: activator ions) [1].

ETU is by far the most efficient upconversion process in RE doped nanomaterials and it is independent of the pump power. In the process of ETU, two situations, resonant non-radiative transfer and phonon-assisted non-radiative transfer in two-ion-involved system, will be mainly discussed (Figure 3) [1]. When the excited energies of sensitizer (S) and activator (A) are nearly equal and the distance between them is near enough, energy can be transferred from S to A, exciting A from its ground state to excited state before S emits photons. In phonon-assisted non-radiative transfer, an energy mismatch exists between S and A ions, so phonon assistance is necessary to have the energy transfer process.

**Figure 4.**
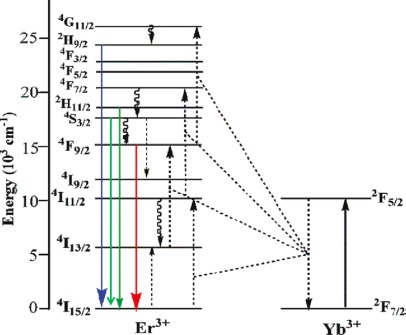
Energy diagram of the Er^3+/^Yb^3+^ codoped materials [24].

For example, in the system of NaYF_4_:Yb^3+^ (Figure 4) Er^3+^ UCNs, red, blue, and green light can be emitted through this process. The Yb^3+^ ion, with its excited state ^2^F_5/2_, has an energy comparable to ^4^I_11/2_ (Er^3+^), which can act as a sensitizer for Er^3+^ and transfer its energy to an unexcited Er^3+^ ion through the energy transfer process: ^2^F_5/2_ (Yb^3+^) + ^4^I_15/2_ (Er^3+^) → ^2^F_7/2_ (Yb^3+^) + ^4^I_11/2_ (Er^3+^). By further cross relaxation and phonon-assisted process, red emission (∼654 nm) can be emitted from ^4^F_9/2_ [^2^F_5/2_ (Yb^3+^) + ^4^I_13/2_ (Er^3+^) → ^2^F_7/2_ (Yb^3+^) + ^4^F_9/2_ (Er^3+^)]. The blue (∼408 nm) and green luminescence emissions (∼526 nm and ∼533 nm) are emitted from^2^H_9/2_ – ^4^I_15/2_ and ^2^H_11/2_ – ^4^I_15/2_ (also ^4^S_3/2_ − ^4^I_15/2_) via similar ways, respectively.

## 3. Synthesis of Rare Earth Fluoride Nanomaterials

In order to obtain high luminescence efficiency, synthesizing high-quality UCNs is very critical. So far, there are three common methods to synthesize UCNs, including thermal decompostition [13,25–30], hydrothermal synthesis [31–33], and ionic liquids-based synthesis [34].

### 3.1. Thermal Decomposition Method

**Figure 5.**
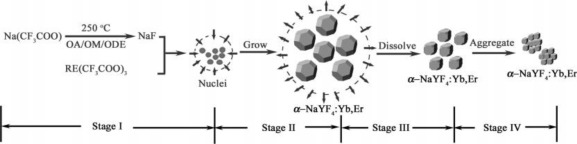
Schematic illustration of the growth stages of α-NaYF_4_:Yb^3+^, Er^3+^ nanocrystals via a delayed nucleation pathway [27].

Thermal decomposition, which gives well shaped particles, with good size control, after a relatively short reaction time, is one of the most popular methods. It usually involves dissolving organic precursors in high-boiling organic solvents with the assistance of surfactants. The commonly used organic precursors are trifluoroacetate compounds, and the surfactants typically have polar capping groups and long hydrocarbon chains, such as oleic acid (OA), omeylamine (OM), and 1-octadecence (ODE). Mai *et al.* [27] systematically investigated the growth mechanism of nanocrystals and pointed out that the trifluoroaetate precursors in hot surfactant solutions went through a unique delayed nucleation pathway. The synthesis reaction was separated as four stages including nucleation in a delayed time, particle growth by monomer supply, size shrinkage by dissolution, and aggregation. Figure 5 illustrates the synthesis steps of α-NaYF_4_:Yb^3+^, Er^3+^ UCNs and by varying the reaction time, concentration of reagents, and reaction temperature, various sizes and shapes of NaYF_4_:Yb^3+^, Er^3+^ UCNs can be obtained.

**Figure 6.**
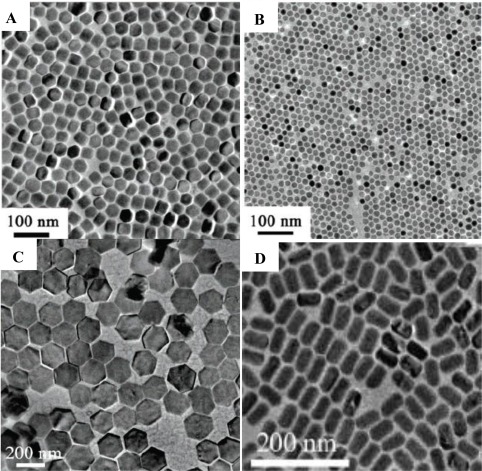
Representative shapes of various RE fluoride nanomaterials by thermal decomposition method [27,28].

Figure 6 shows a few examples of RE fluoride nanomaterials synthesized by thermal decomposition method, which are well-shaped and monodispersed. Although it gives a narrow size distribution and good shape control, the thermal decomposition method still suffers some drawbacks. It normally requires high reaction temperature (250–330 °C), organic solvents, and an oxygen-free with inert gas protection. In addition, most of the synthesized nanomaterials are stabilized by surfactant, which brings difficulties in the biological applications. Further surface modification is also required.

### 3.2. Hydrothermal Synthesis Method

**Figure 7.**
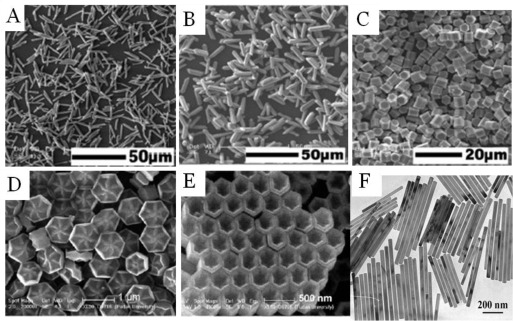
Different shapes of RE fluoride nano-/microcrystals by hydrothermal method [37–39].

Hydrothermal synthesis is a typical solution-based approach, which is usually employed under high temperatures and pressures [5,31,35,36]. Unlike the thermal decomposition method, which can only use an organic compound as a solvent, hydrothermal synthesis can occur in a water-based system and at a lower reaction temperature (160–220 °C) in a relatively environmentally friendly approach. It is an effective and convenient process in preparing inorganic materials with diverse controllable morphologies and architectures. For example, various shapes of hexagonal NaYF_4_ crystals, such as prism, disk, tube, rod, and octadecahedral shapes were synthesized by applying this method [37,38]. Recently, in Lin’s group, the mechanism of synthesizing different shapes of RE fluoride nano-/microcrystals was systematically investigated [33]. It was reported that the organic additive trisodium citrate, the fluoride source, and pH value have great effects on the shapes (Figure 7).

### 3.3. Ionic Liquids-Based Synthesis Method

Compared to the other two methods, ionic liquids-based synthesis is a relatively green method due to not needing organic solvents, low reaction temperature, and a short reaction time. Nanomaterials are synthesized in ionic liquid media, which are known as “green solvents” because of their chemical stability, low vapor pressure, and non-flammability. However, the synthesized nanomaterials are of a lower quality with a broader size distribution, lower monodispersity, and less uniformity when compared with other two methods [40], which limits their applications.

Therefore, the thermal decomposition and hydrothermal synthesis methods are still the most widely used and best methods for producing well-controlled shapes and sizes of RE fluoride nanomaterials.

## 4. Properties and Applications

There has been a growing interest in studying the upconveriosn phenomenon after it was first recognized in the mid-1960s. It has been widely used in solid-state lasers (especially blue-light-emitting lasers) [41], solar cells [42,43], and waveguide amplifiers [44,45]. It was not considered to be used in the biological field until the tremendous advances in nanotechnology over the last decade. The successful synthesis of high-quality, lanthanide-doped upconversion nanomaterials (UCN) have broadened their applications. Nondestructive optical memory [46] and rewritable optical storage [47] applications based on UCNs have been developed recently. Furthermore, these UCNs with their controllable sizes and unique luminescence properties have become prominent in the biological field as promising alternatives to conventional organic dyes and quantum dots (QDs). Compared to organic dyes and QDs, UCNs have high quantum yields, long lifetimes, high photostability, a narrow emission peak, and most importantly, low optical background noise due to the absence of autofluorescence under NIR radiation. Multiple analytes can be detected simultaneously from different UCNs excited by the same IR laser [48]. Furthermore, their applications in biolabeling [6,10,25,49], homogeneous assay [11,50,51], and as reporters for DNA microarrays [52] have been extensively studied. Since these UCNs need to be used in cells or animals, the toxicity study is also very important. Therefore, their viability will now be discussed, followed with their applications in the biological field, including photodynamic therapy, imaging, and others. The recent advances of these UCNs in other fields will be discussed as well.

### 4.1. Toxicity

**Figure 8.**
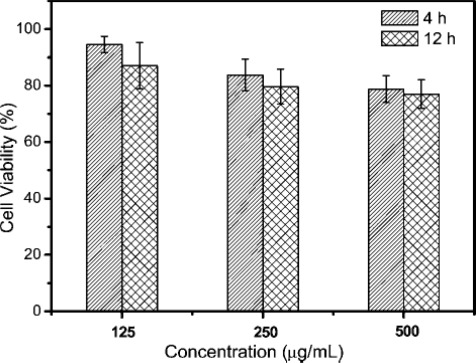
*In vivo* cell viability of KB cells incubated with mPEG-UCNPs at different concentrations for 4–12 h [6].

Due to the rapid progress in developing UCNs’ applications in the biological field, the safety and toxicity of UCNs are a growing concern and extremely important. The most common methods to evaluate the toxicity are through cellular morphology and mitochondrial function, such as methylthiazolyl tetrazolium (MTT) and 3-(4,5-dimethylthiazol-2-yl)-5-(3-carboxymethoxyphenyl)-2-(4-sulfophenyl)-2H-tetrazolium (MTS) assays. Studies have shown that UCNs are non-/low-toxicity to a broad range of cell lines [4,6,10,18,53,54]. The cells were usually treated with different concentrations of UCNs for various time range, and by measuring the cell viability percentage, the UCNs toxicity was determined. For example, in Zhang’s group, they studied both polyethyleneimine (PEI) [55] and silica-coated [18] UCNs’ cell toxicity. In the case of silica-coated UCNs, although the cell viability decreased as a function of both concentration and time, 93.4% of skeletal myoblast cells and 93.2% of BMSCs cells were still alive under 24 h incubation at a concentration of 1 μg/mL, which indicated great biocompatibility [18]. Recently, Li and co-workers investigated the UCNs’ long-term *in vivo* distribution and toxicity [56]. Their findings show that the mice survived for 115 days after the intravenously injection 15 mg/kg of UCNs with no apparent adverse effects observed to their health, indicating the possibility for long-term targeted imaging and therapy studies *in vivo*. Hu *et al.* treated the KB cells with different concentrations of PEG-modificed UCNs (Figure 8) [6]. Even at a high concentration of 250 μg/mL, the cell viability still remained above 80%, showing the low cytotoxicity of the nanoparticles.

### 4.2. Applications in Cancer Therapy

Light has been used to treat various diseases for more than 3,000 years in many countries, such as Ancient Egypt, India, and China [57,58]. More than 100 years ago, Oscar Raab, a German medical student, accidently observed a combination of light and acridine could induce cell death. This was later named as a “photodynamic action” [59]. Photodynamic therapy (PDT) was not approved until 1993 in Canada, where Photofrin was used as a photosensitizer for bladder cancer treatment. A typical PDT process requires three elements: light, photosensitizer, and oxygen. When the photosensitizer is irradiated under certain wavelength, it will be excited from the ground state to the excited state. The energy is released when it returns to the ground state, which is transferred to the nearby oxygen to generate reactive oxygen species (ROS, like singlet oxygen, ^1^O_2_). ROS can cause oxidative damage to nearby cells and ultimately kill the cells [60,61]. PDT, as a cancer therapy, has become a popular and acceptable technique in recent years [62,63] due to a better selectivity for the tumor and a lower systemic toxicity for fewer side effects compared to radiation therapy and chemotherapy [64,65]. Some nanomaterials, such as gold nanomaterials [66–68], QDs [69–73], and polymers [74], have been employed in PDT. However, most of them are through the downconversion luminescence, which needs high energy to activate the photosensitizers. Furthermore, the light needed to activate photosensitizers only can penetrate about a centimeter and can cause normal cell death as well. Their inherent cytoxicity (like QDs), downconversion luminescence property and limited penetration depth all cause hindrances in the further applications in biomedical field. Therefore, it is important to find a better way with high PDT efficiency, deeper penetration, and less side effects.

**Figure 9.**
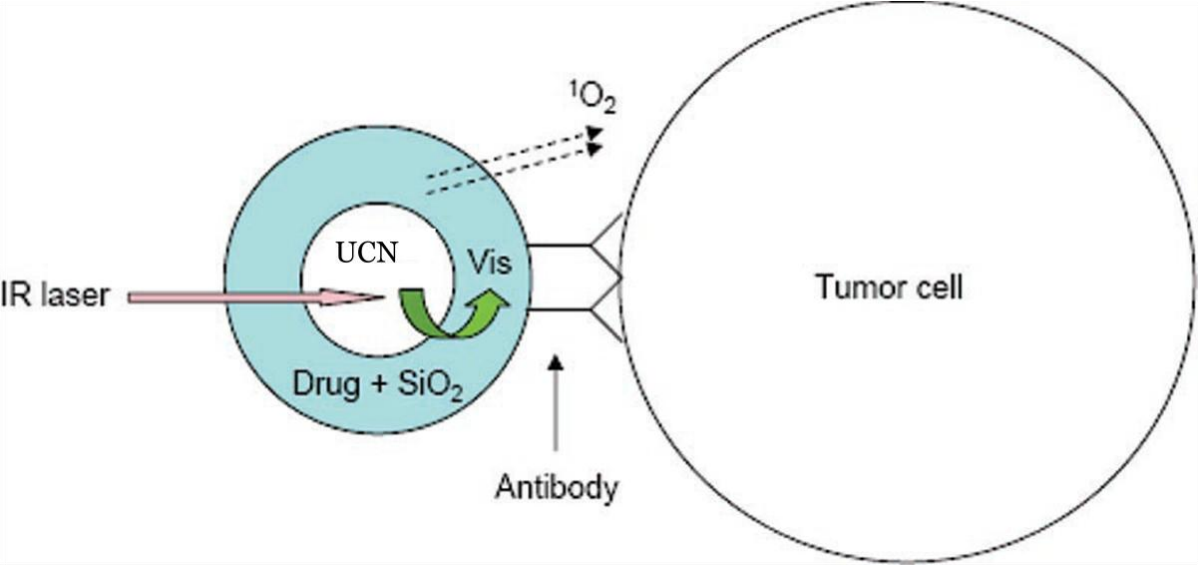
Schematic of the design of the versatile photosensitizer based on UCNs [78].

UCN satisfies all the requirements due to its deeper penetration (capable of converting NIR light into visible light), lower toxicity, higher stability, and easier surface modification. UCNs used in PDT are usually pre-coated with a shell, which has the functions of: (1) doping matrix for photosensitizers; (2) specific target on tumor cells; and (3) UCNs stabilization. The NaYF_4_:Yb/Er UCN is one of the most common used UCN in PDT due to its high UC efficiency [19,75–77]. Zhang and co-workers first demonstrated a novel design for PDT based on UCNs for the treatment of bladder cancer cells (Figure 9) [78]. UCNs were coated with a layer of mesoporous silica shell, in which the photosensitizers were doped. An antibody, which had specific antigens that were expressed on the target cell’s surface, was covalently attached on the surface of the silica shell. Due to the spectra overlap between photosensitizers’ absorbance and UCNs’ emission, the photosensitizer-doped UCNs can generate ^1^O_2_ under NIR irradiation and further kill the target cells. The generation of ^1^O_2_ is usually indicated by the quenching phenomenon of ABDA or ADPA.

Zhang *et al.* applied similar principle to study PDT effect on cancer cells [77]. One thin layer of silica was coated on UCNs, followed by second layer of mesoporous silica, which was further doped with photosensitizers. The photosensitizers doped in the silica matrix can be protected from degradation in the harsh biological environment but the formed singlet oxygen can still be released. The obtained fluorescence image showed the uptake of mesoporous silica coated UCNs by murine bladder cancer cells under the excitation of 980 nm. The significance cell viability difference between photosensitizer-doped UCNs and no-photosensitizer UCNs was observed when both were exposed to NIR laser only for 5 min, indicating the production of singlet oxygen.

### 4.3. Applications in Optical Imaging

Optical imaging has become an essential tool in biological research and clinical application due to its ability of visualizing morphological details in tissue. Although traditional optical imaging has been widely used in *in vivo* and *in vitro* imaging with a combination of different functional nanoparticles, it suffers some inherent limitations such as significant auto-fluorescence from biological tissues, short penetration depth ability, and causing DNA damage or cell death [33,79–83]. These limitations occur because most of them are based on conventional downconversion phenomena, emitting low-energy fluorescence when excited by a high-energy light (usually UV or visible light). Therefore, finding an appropriate alternative, which has low background signal but also deep penetration ability, is very important.

The Ln^3+^-doped materials were first used in tissue imaging in 1999, where Zijlmans *et al.* observed a low autofluorescence signal and no bleaching even after continuous exposure to high excitation energy levels [48]. However, the size of the applied particles was in the level of submicron size, which limited the applications. In recent years, with the rapid development of synthesis techniques, smaller size but with high-quality UCNs are easily obtained. The UCNs-based imaging technique has been explored and widely used in cell, tissue, and animal imaging [12,48,84–89]. Wu and co-workers [90] have recently shown that UCNs can be used for single-molecule imaging and the individual UCNs were bright enough to be imaged with a modest-power CW laser. The high photostability of the UCNs was also observed even after more than 1 h of continuous laser illumination, implying the extraordinary ability of UCNs for long-period observation of cells. Another bioimaging advantage of UCNs is their low background fluorescence and high signal-to-noise ratio.

**Figure 10.**
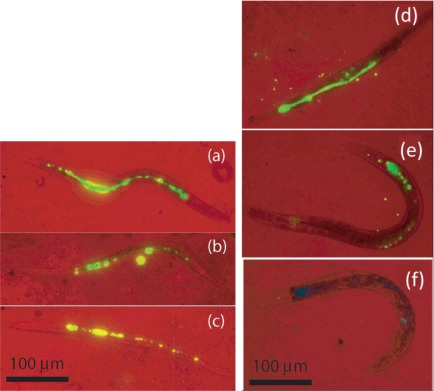
False color two-photon images of *C. elegans* at 980 nm excitation with red representing the bright field and green for the phosphor emission. (**Left**) The worms were deprived of food over a period of 24 h, showing little or no change at (**a**) 0 h, (**b**) 4 h, and (**c**) 24 h. (**Right**) The worms were given food immediately after being fed with phosphors, showing decreasing amounts of phosphors at (**d**) 0 h, (**e**) 1 h, and (**f**) 2 h [22].

Lim *et al.* [22,53] performed *in vivo* and scanning electron microscopy imaging of UCNs in *C. elegans* due to its short life cycle, rapid growth, and appropriate size to optical microscopy. In their study, *C. elegans* were fed with UCNs and imaged at fixed time intervals in order to track the movement of the phosphors through their digestive system. No significant change in the phosphors was monitored up to 24 h and after feeding the worms with food (Figure 10), the phosphors were secreted in under 2 h. It demonstrated that UCNs were nonbleaching, biocompatible, and nontoxic, which make them ideal candidates in the biological system.

**Figure 11.**
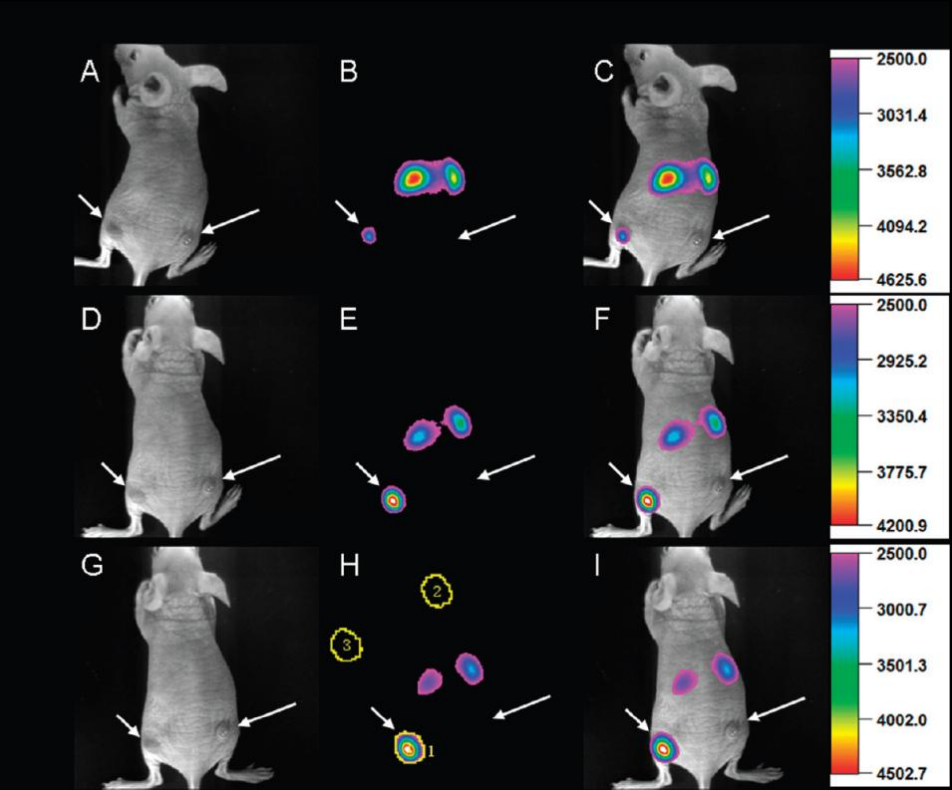
Time-dependent *in vivo* upconversion luminescence imaging of subcutaneous U87MG tumor (left hind leg, indicated by short arrows and MCF-7 tumor (right hind leg, indicated by long arrows) borne by athymic nude mice after intravenous injection of UCN-RGD over a 24 h period. (H) The *in vivo* signal-to-noise ratio (SNR) calculation. Region of interest (ROI)1, specific uptake; ROI 2, nonspecific uptake; ROI 3, background. SNR = (IROI 1 – IROI 3)/(IROI 2 – I ROI 3) [23].

Since Chatterjee *et al.* [55] first studied the *in vivo* imaging of UCNs in small mammals which showed much higher fluorescence compared to QDs, it has made significant progress and attracted great interest. In Li’s group [4], they found a high relaxivity of 5.60 s^−1^ (mM)^−1^ of UCNs, and the UCNs were successfully applied as contrast agents form magnetic resonance imaging (MRI) *in vivo.* The concept of upconversion and magnetic resonance dual-modality imaging *in vivo* of whole-body animals using UCNs with magnetic resonance properties has showed great promise to serve as a platform technology for the next-generation of probes for bioimaging *in vivo*. Fluorescence targeted imaging *in vivo* has proven very useful in tumor recognition and drug delivery. Xiong *et al.* [23] developed a high contrast upconversion imaging protocol based on UCNs as luminescent labels for targeted imaging of tumors both in *in vivo* and *in vitro*. No autofluorescence signal observed in imaging even at high penetration depth (∼600 μm) and the signal-to-noise ratio could be reached ∼24 between the tumor and the background, which cannot be obtained in single-photon or two-photon fluorescence imaging (Figure 11). Their study may open up a new perspective for cell recognition and targeted imaging-guided cancer diagnosis.

**Figure 12.**
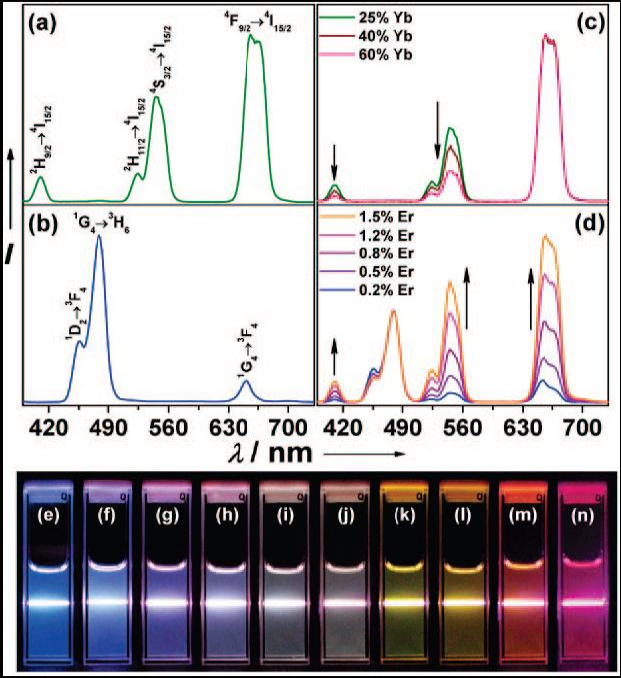
Room temperature upconversion emission spectra of (**a**) NaYF_4_:Yb/Er (18/2 mol%), (**b**) NaYF_4_:Yb/Tm (20/0.2 mol%), (**c**) NaYF_4_:Yb/Er (25–60/2 mol%), and (**d**) NaYF_4_:Yb/Tm/Er (20/0.2/0.2–1.5 mol%) particles in ethanol solutions. The spectra in (c) and (d) were normalized to Er^3+^ 650 nm and Tm^3+^ 480 emissions, respectively. Compiled luminescent photos showing corresponding colloidal solutions of (**e**) NaYF_4_:Yb/Tm (20/0.2 mol%), (**f**–**j**) NaYF_4_:Yb/Tm/Er (20/0.2/0.2–1.5 mol%), and (**k**–**n**) NaYF_4_:Yb/Er (18–60/2 mol%). The samples were excited at 980 nm with a 600 mW diode laser [83].

The ability to manipulate color output of nanomaterials can broaden their applications, especially in the case of multiplexed biological labeling and imaging. Various approaches, based on surface plasmon resonance [91–94] or using multicolor-encoded microbeads and nanoparticles [95,96], have been applied to tune multicolor output. However, most of these methods need high energy excitation source in the UV region, which bring limitations to bioimaging studies due to the significant background (autofluorescence) and photo damage to the samples. The UCNs-based method becomes extremely promising to solve these problems, and a few current researchers have already shown the possibility to output multicolor lights. It can be obtained by adjusting the reaction temperature and time, crystal structure and phase, or changing the combinations of Ln^3+^ dopants and dopant concentration (Figure 12) [26,39,83,97,98]. Ehlert *et al.* presented that four different colors of UCNs can be spectrally separated under multiplexing conditions with a single excitation source of 980 nm [99]. Li and co-workers obtained the multicolor output signal by encapsulating organic dyes or QDs into the silica shell and the upconversion fluorescence was generated based on FRET from the UCN-cores to organic dyes or QDs [100].

The emission efficiency can further be enhanced by combining with other metal nanoparticles [12,101], surface modification [102], and multiple Ln^2+^ ions in the UCNs [103]. Chen *et al.* recently found that by simply increasing the content of the doped Yb^3+^ ions from 20 to 100%, their NIR-to-NIR upconversion photoluminescence intensity increased about 8.6 times per Yb^3+^ concentration and 43 times per nanoparticle [104].

### 4.4. Applications in Sensors

**Figure 13.**
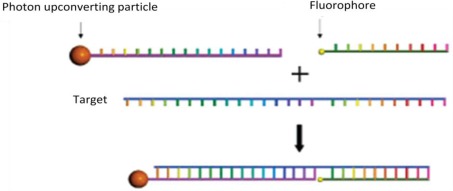
The schematic of the nucleotide sensor design [110].

DNA/RNA analysis is of great importance in molecular biology, genetic, and molecular medicine. Great effects have been invested in the precise concentration detection, such as metal nanoparticle-based analysis [105–108]. In recent years, UCNs were also used for the sensitive detection of oligonucleotides. For example, van de Rijke *et al*. [52] and Corstjens *et al*. [109] used UCNs as direct labeling reagents to detect single strand nucleic acids. A new design of a nucleotide sensor by Zhang *et al*. [110] used UCN as energy donor and the other fluorophore as an energy acceptor in a sandwich assay format (Figure 13). In the presence of UCNs and IR irradiation, the fluorophore was brought close to the UCN and energy transfer took place, leading to the light emission from the fluorophore. The target oligonucleotide can be detected by monitoring the fluorophore emission. This sensor displayed high sensitivity (1.3 nM), high specificity, and self-calibration capability. A general aptasensor for detection of various target molecules was reported recently by Liu *et al.* [111], which was based on UCNs-graphene oxide FRET. This proposed design can be further extended for sensing other kinds of molecules as well as causing structure conformations of ssDNA, which have shown its great potential in clinical diagnostic and biosensing techniques.

The UCNs-based sensors are used not only in the biological field, but also in the chemical area. Mader *et al*. [112] developed an ammonia sensor based on the use of UCNs and the pH probe phenol red immobilized in a polystyrene matrix. In the presence of ammonia, a strong increase in the 560 nm absoption of the pH probe caused the green emission of the UCNs to be screened off, while the red emission of the UCNs remained unaffected. The ratio of the intensities of the green emissions and the red emission of the UCNs served as the detection signal. Achatz *et al*. [113] first developed an oxygen sensor by using UCNs as the nanolamps. In the sensing method they presented, the UCNs were used as light source that can cause photoexcitation of an iridium (III) complex. The fluorescence of this complex was dynamically quenched by oxygen. Most recently, Li *et al*. [114] described a highly selective and sensitive CN^−^ sensor by using the chromophoric iridium (III) complex-coated upconversion nanoparticles. It is the first time that the developed sensor can be used for both bioimaging of an anion in living cells and sensing. This novel CN^−^ sensor can provide a very low detection limit of 0.18 μM in the aqueous solution. Also, a chromophoric rughenium complex-assembled upconversion nanophosphor was developed and used as a Hg^2+^ sensor by Li’s group [115]. The Hg^2+^ nanoprobe showed its capability of not only monitoring the concentration of Hg^2+^ but also monitoring changes in the distribution of Hg^2+^ in living cells by upconversion luminescence bioimaging.

## 5. Conclusions

In this review, the mechanism of upconversion phenomenon, the UCNs’ unique luminescence properties, and the recent advances in UCNs’ chemical synthesis and applications were highlighted. Great progress in size, shape, and phase control of UCNs has been achieved by using various synthesis methods, which also has dramatically broadened their applications in the biological field. In addition to their successful applications in biological labels and fluorescence imaging, they also attracted increasing attention in cancer therapy (mainly focus on PDT). However, there are still some limitations to overcome. First, the synthesized UCNs are normally not water soluble. The one-pot synthesis of water-soluble UCNs always results in broad size distribution and poor shape. Although surface modification can enhance their water solubility and biocompatibility, the procedures are time-consuming and may affect the luminescence efficiency. Secondly, the UCNs-based PDT is still in its infancy. The questions of how to load enough photosensitizers without changing the size significantly, and how to bind the photosensitize-loaded UCNs more specifically to cancer cells still need to be addressed. We believe UCNs-based techniques will be widely exploited and will solve some of today’s most challenging problems.
